# Porcine CD3^+^NKp46^+^ Lymphocytes Have NK-Cell Characteristics and Are Present in Increased Frequencies in the Lungs of Influenza-Infected Animals

**DOI:** 10.3389/fimmu.2016.00263

**Published:** 2016-07-14

**Authors:** Kerstin H. Mair, Maria Stadler, Stephanie C. Talker, Hilde Forberg, Anne K. Storset, Andrea Müllebner, J. Catharina Duvigneau, Sabine E. Hammer, Armin Saalmüller, Wilhelm Gerner

**Affiliations:** ^1^Department of Pathobiology, Institute of Immunology, University of Veterinary Medicine Vienna, Vienna, Austria; ^2^Department of Laboratory Services, Norwegian Veterinary Institute, Oslo, Norway; ^3^Department of Food Safety and Infection Biology, Norwegian University of Life Sciences, Oslo, Norway; ^4^Department of Biomedical Sciences, Institute of Medical Biochemistry, University of Veterinary Medicine Vienna, Vienna, Austria

**Keywords:** NKp46, CD3, swine, NK cells, influenza

## Abstract

The CD3^−^NKp46^+^ phenotype is frequently used for the identification of natural killer (NK) cells in various mammalian species. Recently, NKp46 expression was analyzed in more detail in swine. It could be shown that besides CD3^−^NKp46^+^ lymphocytes, a small but distinct population of CD3^+^NKp46^+^ cells exists. In this study, we report low frequencies of CD3^+^NKp46^+^ lymphocytes in blood, lymph nodes, and spleen, but increased frequencies in non-lymphatic organs, like liver and lung. Phenotypic analyses showed that the majority of CD3^+^NKp46^+^ cells coexpressed the CD8αβ heterodimer, while a minor subset expressed the TCR-γδ, which was associated with a CD8αα^+^ phenotype. Despite these T-cell associated receptors, the majority of CD3^+^NKp46^+^ lymphocytes displayed a NK-related phenotype (CD2^+^CD5^−^CD6^−^CD16^+^perforin^+^) and expressed mRNA of NKp30, NKp44, and NKG2D at similar levels as NK cells. Functional tests showed that CD3^+^NKp46^+^ lymphocytes produced IFN-γ and proliferated upon cytokine stimulation to a similar extent as NK cells, but did not respond to the T-cell mitogen, ConA. Likewise, CD3^+^NKp46^+^ cells killed K562 cells with an efficiency comparable to NK cells. Cross-linking of NKp46 and CD3 led to degranulation of CD3^+^NKp46^+^ cells, indicating functional signaling pathways for both receptors. Additionally, influenza A(H1N1)pdm09-infected pigs had reduced frequencies of CD3^+^NKp46^+^ lymphocytes in blood, but increased frequencies in the lung in the early phase of infection. Thus, CD3^+^NKp46^+^ cells appear to be involved in the early phase of influenza infections. In summary, we describe a lymphocyte population in swine with a mixed phenotype of NK and T cells, with results so far indicating that this cell population functionally resembles NK cells.

## Introduction

The activating receptor NKp46 (NCR1, CD335) belongs to the family of natural cytotoxicity receptors (NCRs) and was initially described to be specifically expressed on natural killer (NK) cells (1–3). Receptor triggering on NK cells results in the induction of cytokine production like IFN-γ and in cytolytic activity (1, 4). The receptor recognizes hemagglutinins (HA) of influenza, parainfluenza, and Sendai virus, and ligation leads to lysis of infected cells (5–7). Likewise, recognition of currently unknown ligands on transformed cells induces killing of tumor cells (6, 8). Therefore, NKp46 is involved in the defense of viral infections and cancer.

NKp46 is detectable early in NK-cell development and expression is maintained in later developmental stages (9, 10). Furthermore, NKp46 seems to be evolutionarily conserved in mammals and, therefore, was proposed to be a general marker to define NK cells across mammalian species (9, 11). Hence, NKp46 expression was used to identify and characterize NK cells in humans (1, 2), rodents (3, 9, 12), cattle (13), sheep (14), and pig (15). For swine, three distinct NK-cell subsets could be identified: NKp46^−^, NKp46^+^, and NKp46^high^ CD3^−^ lymphocytes that display phenotypic and functional properties of NK cells (15, 16). It was also shown that porcine NKp46 binds to hemagglutinin of influenza virus and that NKp46^+^ lymphocytes accumulate in lungs of H1N1 influenza virus-infected animals (17).

Moreover, it became evident that NKp46 is also expressed on a minor fraction of different T-cell populations. In mouse, rare subsets of NKp46^+^ cells coexpressing CD3 could be identified (9, 10). Likewise, in humans, the existence of CD3^+^NKp46^+^ cells in lymphatic and non-lymphatic tissues could be demonstrated (18). Lymphocytes with this phenotype could be identified within the γδ T-cell population in mice (19) and in NKT-cell subsets in mouse and human (20, 21). Although these cells account for only a minute fraction of lymphocytes, it was observed that the NKp46^+^ NKT cells expand during leukemic transformation (20) and viral infection (21). Additionally, it could be shown that NKp46 is induced in cytolytic T cells by chronic activation of cells during autoimmune diseases (22). In cattle, it could be shown that γδ T cells from blood and spleen express NKp46 after prolonged *in vitro* stimulation with IL-15 (23). Furthermore, a population of bovine CD3^+^NKp46^+^ lymphocytes has been described that represents a non-conventional T-cell subset that is constitutively present in the blood of healthy cattle (24). Likewise, in the dog, a CD3^+^NKp46^+^ lymphocyte subset could be identified in 79% of animals analyzed (25).

A distinct population of CD3^+^NKp46^+^ cells could also be identified in the pig (15). To further investigate this lymphocyte population in more detail, we performed phenotypic and functional studies on porcine CD3^+^NKp46^+^ lymphocytes and compared them with NK and T cells. We, here, report that the majority of CD3^+^NKp46^+^ cells express the CD8αβ heterodimer, comparable to porcine cytolytic T cells, while a minor subset belongs to TCR-γδ^+^ T cells. Nonetheless, CD3^+^NKp46^+^ cells express NK-associated molecules, such as perforin, CD16, NKp30, and NKp44. Functionally, they respond to *in vitro* stimulation in a NK-like manner and have the capacity of spontaneous cytolytic activity. Degranulation could be induced in CD3^+^NKp46^+^ lymphocytes by receptor triggering of both NKp46 and CD3. Furthermore, we show that CD3^+^NKp46^+^ lymphocytes are present in increased frequencies in lungs of influenza-infected animals in the early phase of infection.

## Materials and Methods

### Isolation of Porcine Lymphocytes

Blood and organs were obtained from healthy 3- to 7-month-old pigs from an abattoir or from animals housed at the University Clinic for Swine at the University of Veterinary Medicine Vienna, Austria. Animals from the slaughterhouse were subjected to electric high-voltage anesthesia followed by exsanguination, a procedure that is in accordance to the Austrian Animal Welfare Slaughter Regulation. In-house pigs were anesthetized by intramuscular injection of Ketaminhydrochlorid (Narketan^®^, Vétoquinol, Vienna, Austria, 10 mg/kg body weight) and Azaperon (Stresnil^®^, Janssen Pharmaceutica, Beerse, Belgium, 1.3 mg/kg body weight). Subsequently, animals were euthanized *via* intracardial injection of T61^®^ (MSD Animal Health, Vienna, Austria, 1.0 ml/10 kg body weight). This procedure was approved by the institutional ethics committee and the national authority according to §26 of Law for Animal experiments, Tierversuchsgesetz 2012 – TVG 2012 (reference number bmwf GZ 68.205/0103-II/3b/2013).

PBMC were isolated from heparinized blood using density gradient centrifugation (Pancoll human, density: 1.077 g/ml, PAN-Biotech, Aidenbach, Germany). Dissected spleens and mediastinal lymph nodes were cut into small pieces and mechanically dissociated by a sieve. Obtained spleen cells were applied to density gradient centrifugation. Isolated cells from lymph nodes were applied to cotton wool filtration to remove dead cells. Lymphocytes from lung tissue were isolated, as described elsewhere (17). Briefly, lung tissue was cut in small pieces and incubated for 1 h at 37°C in cell culture medium containing 2% FCS (PAA, Pasching, Austria), 20 mM Hepes (Sigma-Aldrich, Vienna, Austria), 25 U/ml DNase I (Life Technologies, Carlsbad, CA, USA), and 300 U/ml Collagenase type I (Life Technologies). The cell suspension was subsequently applied to cotton wool filtration and density gradient centrifugation.

Isolated cells from PBMC and organs were either immediately used for phenotypic analyses or stored at −150°C. When frozen cells were used for short-term functional assays, PBMC were thawed 1 day prior to stimulation and rested overnight in culture medium.

### Cell Culture

The human leukemia cell line K562 (26) and isolated porcine PBMC were propagated in RPMI 1640 with stable glutamine (PAN Biotech) supplemented with 10% (v/v) heat-inactivated FCS (PAA), 100 IU/ml penicillin, and 0.1 mg/ml streptomycin (PAA). Cell culture medium for sorted cell subsets was additionally supplemented with 1 mM sodium pyruvate (PAA), non-essential amino acids (PAA), and 50 μM 2-mercaptoethanol (Sigma-Aldrich).

### Flow Cytometry and Antibodies

For flow cytometric (FCM) analyses, cells were either re-suspended in PBS-based buffer containing 10% (v/v) porcine plasma for direct analysis after isolation or in buffer containing 3% (v/v) FCS for analysis after *in vitro* cultivation. All incubation steps were performed in 96-well round-bottom plates at 4°C for 20 min. The different combinations of primary monoclonal antibodies (mAbs) and secondary reagents used for each assay are listed in Table 1. Non-commercial antibodies were produced in-house (27). Where indicated, these antibodies were conjugated either to fluorochromes or Biotin. Alexa Fluor-647 or Alexa Fluor-488 Labeling Kits (Life Technologies) were used according to manufacturer’s instructions. FITC conjugation was performed as described elsewhere (28). Sulfo-NHS-LC Biotin (Thermo Scientific, Pierce, Vienna, Austria) was used for the biotinylation reaction according to manufacturer’s protocol. If unlabeled and directly conjugated antibodies with the same isotype were used in combination, a sequential staining was performed. After labeling with unconjugated primary mAbs and isotype-specific dye-conjugated secondary antibodies, free binding sites were blocked by whole mouse IgG molecules (2 μg per sample, Jackson ImmunoResearch, Suffolk, UK). Thereafter, cells were incubated with directly labeled primary mAbs. For exclusion of dead cells, Fixable Near-IR Dead Cell Stain Kit (Life Technologies) was used according to manufacturer’s protocol with 0.05 μl reactive dye per sample. Appropriate isotype-matched control antibodies were used to assess unspecific bindings. Single-color samples were prepared for automatic compensation.

**Table 1:** **Primary antibodies and secondary reagents used for FCM analyses**.

Antigen	Clone	Isotype	Fluorochrome	Labeling strategy	Source of primary Ab
**Phenotyping of isolated cells**					
CD2	MSA4	IgG2a	Alexa488	Secondary antibody^a^	In-house
CD3	PPT3	IgG1	BV421	Biotin-streptavidin^b^	Southern Biotech
CD3	BB23-8E6-8C8	IgG2a	PerCP-Cy5.5	Directly conjugated	BD Biosciences
CD5	b53b7	IgG1	Alexa488	Secondary antibody^c^	In-house
CD6	a38b2	IgG1	Alexa488	Secondary antibody^c^	In-house
CD8α	76-2-11	IgG2a	Alexa488 or PE-Cy7	Secondary antibody^a^/^d^	In-house
CD8β	PPT23	IgG1	PE	Secondary antibody^e^	In-house
CD8β	PG164A	IgG2a	PE	Secondary antibody^f^	VMRD^k^
CD8β	PPT23	IgG1	Alexa488	Directly conjugated	In-house
CD16	G7	IgG1	Alexa488	Secondary antibody^c^	AbD Serotec^l^
CD27	b30c7	IgG1	BV421	Biotin-streptavidin^b^	In-house
TCR-γδ	PPT16	IgG2b	BV421	Biotin-streptavidin^b^	In-house
SLA-DR	MSA3	IgG2a	Alexa488	Secondary antibody^a^	In-house
NKp46	VIV-KM1	IgG1	Alexa647	Directly conjugated	In-house
Perforin	δG9	IgG2b	PE	Directly conjugated	eBioscience^m^
**FACS sort**					
CD3	BB23-8E6	IgG2b	Alexa488	Secondary antibody^g^	Southern Biotech
CD8α	11/295/33	IgG2a	Alexa647	Secondary antibody^h^	In-house
NKp46	VIV-KM1	IgG1	PE	Secondary antibody^e^	In-house
**Intracellular cytokine staining**					
CD3	BB23-8E6-8C8	IgG2a	PerCP-Cy5.5	Directly conjugated	BD Biosciences
CD8α	11/295/33	IgG2a	FITC	Directly conjugated	In-house
CD8β	PPT23	IgG1	BV605	Biotin-streptavidin^i^,^j^	In-house
NKp46	VIV-KM1	IgG1	Alexa647	Directly conjugated	In-house
IFN-γ	P2G10	IgG1	PE	Directly conjugated	BD Biosciences
**Violet proliferation assay**					
CD3	BB23-8E6-8C8	IgG2a	PerCP-Cy5.5	Directly conjugated	BD Biosciences
CD8α	76-2-11	IgG2a	PE	Directly conjugated	BD Biosciences
CD8β	PPT23	IgG1	BV605	Biotin-streptavidin^i^,^j^	In-house
CD16	G7	IgG1	FITC	Directly conjugated	AbD Serotec
NKp46	VIV-KM1	IgG1	Alexa647	Directly conjugated	In-house
**CD107a degranulation assay**					
CD3	BB23-8E6-8C8	IgG2a	PerCP-Cy5.5	Directly conjugated	BD Biosciences
CD8α	76-2-11	IgG2a	PE	Directly conjugated	BD Biosciences
NKp46	VIV-KM1	IgG1	Alexa647	Directly conjugated	In-house
CD107a	4E9/11	IgG1	FITC	Directly conjugated	AbD Serotec

*^a^goat anti-mouse anti-IgG2a-Alexa488, Life Technologies*.

*^b^Streptavidin-Brilliant Violet 421, BioLegend, San Jose, CA, USA*.

*^c^goat anti-mouse anti-IgG1-Alexa488, Life Technologies*.

*^d^goat anti-mouse anti-IgG2a-PE-Cy7, Southern Biotech*.

*^e^goat anti-mouse anti-IgG1-PE, Southern Biotech, Birmingham, AL, USA*.

*^f^goat anti-mouse anti-IgG2a-PE, Southern Biotech*.

*^g^goat anti-mouse anti-IgG2b-Alexa488, Life Technologies*.

*^h^goat anti-mouse anti-IgG2a-Alexa647, Life Technologies*.

*^i^goat anti-mouse anti-IgG1-Biotin, Southern Biotech*.

*^j^Streptavidin-Brilliant Violet 605, BioLegend*.

*^k^VMRD, Pullman, WA, USA*.

*^l^AbD Serotec, Raleigh, NC, USA*.

*^m^eBioscience, San Diego, CA, USA*.

All samples containing freshly isolated cells were treated with a fixation and permeabilization reagent prior to analyses, to lyse remaining erythrocytes (BD Cytofix/Cytoperm^™^ Fixation/Permeabilization Kit, BD Biosciences, San Jose, CA, USA). Where applicable, this was followed by an incubation step with directly labeled mAbs against intracellular antigens. For intracellular staining of perforin, cells were fixed and permeabilized with in-house made saponin-containing reagents as described elsewhere (29).

Flow cytometric analyses were performed on a FACSCanto II equipped with a high throughput sampler (BD Biosciences). At least 2 × 10^5^ live lymphocytes were recorded per sample. Data were analyzed with FACSDiva software (Version 6.1.3., BD Biosciences) and FlowJo software (Version 7.6.3., Tree Star, Ashland, OR, USA).

### Fluorescence-Activated Cell Sorting

PBMC were labeled with primary antibodies against CD3, CD8α, and NKp46, as well as corresponding secondary reagents as described in Table 1. PBS containing 5% FCS (v/v) and 2 mM EDTA was used for all washing steps. PBMC were sorted into CD3^+^NKp46^−^ T cells, CD3^−^CD8α^+/dim^ total NK cells, and CD3^+^NKp46^+^ lymphocytes on a FACSAria (BD Biosciences). All sorted subsets had a purity of 97.0% or higher. After two washing steps in cell culture medium, sorted cells were cultivated for functional assays. Alternatively, sorted cells were thoroughly washed with PBS and cell pellets were snap-frozen in liquid nitrogen and stored at −80°C for subsequent mRNA analysis.

### Intracellular Cytokine Staining for IFN-γ

Total PBMC were cultivated in 96-well round-bottom plates at 3 × 10^5^ cells per well in a final volume of 200 μl. Cells were incubated in the presence of phorbol-12-myristate-13-acetate (PMA, 50 ng/ml) and Ionomycin (500 ng/ml, both Sigma-Aldrich) for 4 h. Alternatively, cells were stimulated with a combination of recombinant porcine (rp) IL-2 (20 ng/ml), rpIL-12 (25 ng/ml), and rpIL-18 (two different concentrations: 5 ng/ml and 25 ng/ml) for 24 h (all cytokines from R&D Systems, Minneapolis, MN, USA). Cells cultured in medium alone served as negative control. For intracellular cytokine staining, Brefeldin A (GolgiPlug, BD Biosciences) was added at a final concentration of 1 μg/ml per well 4 h prior to harvest. Subsequently, cells were subjected to FCM staining as outlined in Table 1.

### Proliferation Assay

Total PBMC were stained with CellTrace^™^ Violet Proliferation Kit (Life Technologies) following manufacturer’s instructions, as described elsewhere (30). Briefly, cells were washed in PBS and adjusted to 5–20 × 10^6^ cells per ml, followed by addition of 1 ml of a 5 μM solution of the Violet Cell Trace dye. Labeling was performed for 10 min at 37°C, followed by addition of 2 ml FCS and further incubation for 15 min at room temperature. After three washing steps, cells were cultivated in 96-well round-bottom plates at 3 × 10^5^ cells per well in a final volume of 200 μl for 4 days. Cells were either stimulated with ConA (3 μg/ml, Amersham Biosciences, Uppsala, Sweden), a combination of rpIL-2 (50 ng/ml, R&D Systems) and rpIL-15 (50 ng/ml, Invitrogen, Carlsbad, CA, USA) or left in medium alone as negative control. Following stimulation, cells were harvested and subjected to FCM staining as outlined in Table 1.

### Cytotoxicity Assay

Fluorescence-activated cell sorting (FACS)-sorted T- and NK-cell populations were used in a 4-h cytotoxic assay as described elsewhere (31). Briefly, sorted cells were plated in 96-well round-bottom plates at the final cell numbers of 20 × 10^4^, 10 × 10^4^, 5 × 10^4^, 2.5 × 10^4^, and 1.25 × 10^4^ and stimulated with rpIL-2 (20 ng/ml, R&D Systems) and rpIL-15 (20 ng/ml, Invitrogen) for 36 h. After pre-activation by cytokines, K562 cells labeled with DIOC_18_ (Sigma-Aldrich) were added to the effector cells with 1 × 10^4^ cells per well. After 4 h, cells were harvested and 30 μl of a 20 μg/ml propidium iodide solution (Sigma-Aldrich) was added per sample prior to FCM analysis for identification of dead cells. At least 5 × 10^3^ DIOC-positive cells were recorded per sample. Percent specific lysis was calculated with the formula: (% of target cell lysis in the test − % of spontaneous cell death)/(% of maximum lysis − % of spontaneous cell death) × 100. For determination of maximum lysis, target cells were fixed and permeabilized with in-house made saponin-containing reagents (29).

### Stimulation of Lymphocytes by Receptor Triggering

Receptor triggering was performed by using mAbs against NKp46 (IgG1, clone VIV-KM3) and CD3 (IgG1, clone PPT7). Isotype-matched irrelevant antibodies (IgG1, clone NCG01, Dianova, Hamburg, Germany) served as control. 96-well round-bottom plates were coated with mAbs at a concentration of 3 μg/ml in PBS (50 μl per well) overnight at 4°C. Plates were washed three times with PBS prior to addition of cells.

Cells used in IFN-γ and CD107a degranulation assays were pre-activated with cytokines, prior to functional tests. For IFN-γ production, PBMC were stimulated with rpIL-2 (25 ng/ml) and IL-18 (5 ng/ml, both R&D Systems). For the degranulation assay, cells were pre-incubated with rpIL-2 (10 ng/ml, R&D Systems) and rpIL-15 (10 ng/ml, Invitrogen) for 24 h. After pre-activation, cells were transferred at 3 × 10^5^ cells in a total volume of 200 μl per well into the mAb-coated plates. Cells were cultured for 4 h together with Brefeldin A (GolgiPlug, BD Biosciences; 1 μg/ml) and Monensin (GolgiStop, BD Biosciences; 2 μg/ml). For the degranulation assay, microcultures were additionally supplemented with FITC-conjugated anti-CD107a mAb at a concentration of 4 μg/ml.

For proliferation assays, PBMC were stained with Violet CellTrace™ dye as stated above. Cells were added to mAb-coated plates at 3 × 10^5^ cells in a total volume of 200 μl per well and incubated for 4 days in cell culture medium without further supplements.

After harvesting, cells were forwarded to FCM analysis. Cells were stained with mAb-combinations of the respective functional assay, as described in Table 1, with the exception of anti-CD8β mAbs that were not included in these analyses. Since NKp46 and CD3 were readily internalized after receptor triggering, detection of these two markers by FCM was improved by an additional staining step with CD3- and NKp46-specific mAbs after permeabilization of cells with BD Cytofix/Cytoperm^™^ Fixation/Permeabilization Kit.

### Analysis of Gene Expression by Quantitative Reverse-Transcriptase PCR

Total RNA of FACS-sorted lymphocyte subsets from blood was isolated using TRI Reagent (Sigma-Aldrich) according to manufacturer’s protocol. RNA quality control and cDNA synthesis were performed as described elsewhere (32). Expression of the target genes NKp30, NKp44, and NKG2D was determined by real-time PCR. Primers and assay setup for NKG2D was performed, as described previously (16). Information on primers is listed in Table 2.

**Table 2:** **Primers used in the RT-qPCR assays of sorted lymphocyte subsets**.

Gene accession no.	Primer and probe sequences: forward (F), reverse (R) and probe (P)	Position on + strand	Product length (bp)
**NKp30**			
XM_003128312.2	**F:** CGGATGCTGTTGCTCATCTT	7	140
	**R:** GCCAATCTCCTCTGGCTGG	146	
**NKp44**			
XM_013977918.1	**F:** TCCGTGAGGTTCCATCTGGCCGTGT	590	140
	**R:** TGTGAAAGGGCAGCGATGGCAGAGG	729	
**NKG2D**			
NM_213813.2	**F:** ACAGCAGAGAAGACCAGGATTTCTTCA	408	104
	**R:** GGAACCATCTTCCCACTGCCAGG	511	
**Cyclophilin A**			
NM_214353.1	**F:** TGCTTTCACAGAATAATTCCAGGATTTA	158	77
	**R:** GACTTGCCACCAGTGCCATTA	234	
	**P: TR-**TGCCAGGGTGGTGACTTCACACGCC**-BHQ2**	188	
**GAPDH**			
NM_001206359.1	**F:** ACATGGCCTCCAAGGAGTAAGA	1083	106
	**R:** GATCGAGTTGGGGCTGTGACT	1188	
	**P: HEX-**CCACCAACCCCAGCAAGAGCACGC**-BHQ1**	1114	

*Sequences of primers (5′–3′) for target genes and reference genes, primer positions on (+) strand, and length of specific product in base pairs (bp) are indicated*.

*For reference genes also sequences of corresponding probes are included*.

For amplification of target genes, SYBR^®^ green I (0.5×, Sigma-Aldrich) was used as reporter dye. The qPCR reaction-mixes contained iTaq^®^ DNA polymerase (0.3 U/reaction, Bio-Rad, Hercules, CA, USA), gene specific primers (250 nmol/l each), a final concentration of 200 μmol/l dNTP each, and 3 mmol/l MgCl_2_ for NKG2D and 1.5 mmol/l MgCl_2_ for NKp30 and NKp44 within the reaction buffer (1×, Bio-Rad). PCR conditions as well as optimization and validation of the qPCR assays are described in more detail in the Supplementary Material. The reference genes Cyclophilin A and GAPDH were measured, as described previously (33), and used to normalize each target-gene expression. Corresponding RT-minus controls and the no-template controls were included in each assay. All samples were measured in duplicates. Quantitative PCR was performed on a CFX96^™^ (Bio-Rad), and data were analyzed using the CFX manager software (Bio-Rad) in the linear regression mode. Target gene expression was displayed as 2^−ΔΔCq^ values representing the fold changes relative to the mean value of CD3^+^NKp46^+^ cells set to a value of 1.

### Influenza Infection Study

Influenza infection studies were carried out at the Animal Health and Veterinary Laboratories Agency, Addlestone, UK. The project was approved by the Animal Health and Veterinary Laboratories Agency Ethics Committee and all procedures were conducted in accordance with the UK Animals (Scientific Procedures) Act 1986 under Project License Permit Number 70/7062. Detailed descriptions on infection procedure and performed analyses are described elsewhere (17). Briefly, 12 six-week-old healthy and influenza-free large white cross-bred pigs were experimentally infected with influenza A/Hamburg/05/2009-e 95 (Ham-e) *via* the intranasal route with 10^6^ TCID_50_ in 2 ml per nostril using a mucosal atomization device (MAD 300, Wolfe Tory Medical). In parallel, nine pigs served as control group, of which six animals were euthanized during the study and three animals were used for blood analysis only. On days 1–3 post-infection (p.i.), four infected and two control pigs were euthanized daily.

Heparinized blood was collected from all animals on each sampling day. Samples from lung tissue were taken postmortem from the cranial, middle, caudal, and accessory lobes of the right lung. PBMC and lymphocytes from lung tissue were isolated, as described earlier, and analyzed by flow cytometry. The following primary and secondary antibodies were used: CD3-eFluor450 (IgG1, clone PPT3), CD8α (IgG2a, clone 11/295/33, unconjugated or AlexaFluor 647 conjugated), NKp46-biotin (IgG1, clone VIV-KM1), goat anti-mouse IgG2a-AlexaFluor 488 (Life Technologies), Streptavidin-AlexaFluor 647 (Life Technologies), and Streptavidin-PE (eBioscience). For intracellular staining, cells were fixed and permeabilized using the BD Cytofix/Cytoperm Fixation/Permeabilization Solution Kit (BD Biosciences), followed by intracellular staining with Ki-67-FITC mAbs (IgG1, clone B56, BD Biosciences). Appropriate secondary and isotype controls were included. Dead cells were excluded using Fixable Near-IR Dead Cell Stain Kit (Life Technologies) according to manufacturer’s protocol. Acquisition was performed on a MACSQuant analyser flow cytometer (Miltenyi Biotec) and data were analyzed using MACSQuantify software. A total of 1 × 10^5^ cells were analyzed when possible. Data from one control animal had to be excluded in the Ki-67 staining of lung lymphocytes, due to insufficient cell numbers.

### Statistical Analysis

Data were analyzed for statistical significance by SPSS^®^ (SPSS Statistics Version 20.0, IBM Corp., Armonk, NY, USA). Obtained values were verified for normal distribution by the Kolmogorov–Smirnov test. Where required, data sets were previously subjected to log-transformation to meet the condition of normality. Data obtained from sets with three or more groups were compared individually by paired two-tailed Student’s *t*-test to the CD3^+^NKp46^+^ subset only. No statistical analyses were performed between other cell subsets within these data sets. Box-plots were created by SigmaPlot software (Version 11.0, Systat Software Inc., Erkrath, Germany). For data of the influenza infection study, box-plots were created by Graph Pad Prism V6 (GraphPad Software), statistical analyses were performed in JMP V9 (SAS Institute, Inc.) using the Mann–Whitney *U* test, as described previously (17). Levels of significance were defined as: *p ≤ 0.05, **p ≤ 0.01, and ***p ≤ 0.001.

## Results

### Identification of Porcine NKp46^+^ Lymphocytes Coexpressing CD3

**Figure 1:**
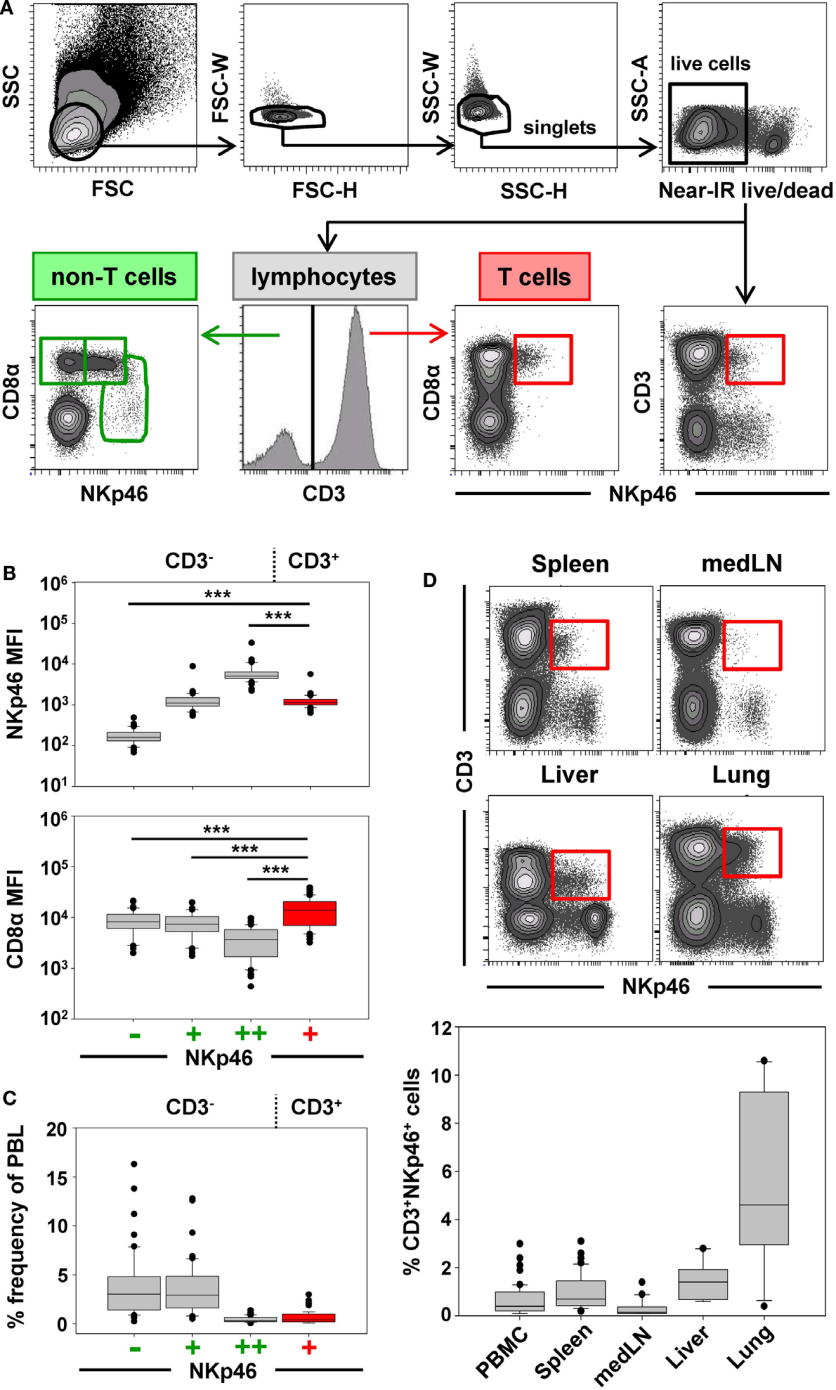
**Porcine CD3^+^NKp46^+^ lymphocytes in lymphatic and non-lymphatic organs**. **(A)** Lymphocytes were gated according to their light scatter properties. Potential doublet cells were excluded by consecutive FSC-H/FSC-W and SSC-H/SSC-W gates, followed by gating of Near-IR^−^ cells to exclude dead cells. Live lymphocytes were separated into CD3^−^ non-T cells to analyze CD8α/NKp46-defined NK-cell subsets (CD8α^+^NKp46^−^, CD8α^+^NKp46^+^ and CD8α^dim/−^NKp46^high^, green gates on the left) and CD3^+^ T cells to analyze CD3^+^NKp46^+^ lymphocytes (red gate on the right). The gating strategy is shown as a representative example for PBMC and was performed correspondingly for all organs analyzed. **(B)** The three CD8α/NKp46-defined NK-cell subsets as well as the CD3^+^NKp46^+^ lymphocytes (shown in red) in blood of 50 individuals were analyzed for their NKp46 (top) and CD8α (bottom) expression levels. Box-plots show the median fluorescence intensities of both markers within respective subsets. Significant differences between the CD3^+^NKp46^+^ cells and NK-cell subsets are indicated (***p ≤ 0.001). **(C)** Box-plots show the frequencies of the four CD3/CD8α/NKp46-defined lymphocyte subsets in blood (*n* = 50). CD3^+^NKp46^+^ cells are indicated in red. **(D)** CD3^+^NKp46^+^ cells (red gates) were analyzed in lymphocytes isolated from blood (*n* = 50), spleen (*n* = 40), mediastinal lymph node (medLN, *n* = 20), liver (*n* = 10), and lung (*n* = 10). Representative examples of one animal are shown in the contour-plots. Box-plots show frequencies of CD3^+^NKp46^+^ lymphocytes within respective organs. All results were obtained from healthy 3- to 7-month-old pigs.

Previous studies on NKp46^+^ cells in swine had identified a small but distinct population of lymphocytes that coexpressed NKp46 and CD3 (15). Since these cells phenotypically seem to be positioned at the interface between NK cells and T cells, we aimed to investigate this lymphocyte subset in more detail. Therefore, FCM analyses were performed with lymphocytes isolated from blood as well as lymphatic and non-lymphatic organs. A uniform gating hierarchy was used throughout all experiments (Figure 1A). Doublet cells and dead cells were excluded, and live lymphocytes were divided upon their CD3 expression. CD3^−^ “non-T cells” were further subgated according to their CD8α/NKp46 expression into the three established porcine NK-cell subsets: CD8α^+^NKp46^−^, CD8α^+^NKp46^+^, and CD8α^dim/−^NKp46^high^ (Figure 1A, bottom row on the left). Additionally, CD3^+^ T cells were analyzed for NKp46.

CD3^+^NKp46^+^ lymphocytes were analyzed in PBMC of 50 healthy 3- to 7-month-old pigs and compared with the three NKp46-defined NK-cell subsets in regard to their NKp46 and CD8α expression levels (Figure 1B). CD3^+^NKp46^+^ cells displayed intermediate expression levels of NKp46, with a median fluorescence intensity (MFI) comparable to the CD3^−^NKp46^+^ NK-cell subset (CD3^+^: 1429 ± 702, CD3^−^: 1368 ± 1148, Figure 1B, upper graph). However, CD8α expression was significantly higher in the CD3^+^NKp46^+^ cells (MFI 18,015 ± 10,129) compared with all three NK-cell subsets (NKp46^−^: MFI 10,195 ± 5063, NKp46^+^: MFI 9246 ± 4825, NKp46^high^: MFI 4724 ± 3077, Figure 1B, bottom graph). Therefore, the CD8α expression level of CD3^+^NKp46^+^ lymphocytes resembles the CD8α^high^ phenotype of CD4^−^TCR-αβ^+^ cytolytic T cells in swine (34, 35).

Although the CD3^+^NKp46^+^ subset could be identified in the blood of all animals analyzed, these cells represented a small lymphocyte population (0.1–3.0%, Figure 1C). This low abundance was comparable to the frequency of the CD8α^dim/−^ NKp46^high^ NK-cell subset in blood (0.1–1.7%). Since it could be shown that the NKp46-defined NK-cell subsets show different homing preferences in the pig (15, 16), different lymphatic and non-lymphatic organs were analyzed for the frequencies of the CD3^+^NKp46^+^ lymphocytes (Figure 1D). In lymphatic organs, the proportion of CD3^+^NKp46^+^ lymphocytes was only slightly higher for spleen (0.2–3.1%) compared with blood (0.1–3.0%), whereas numbers were lower in mediastinal lymph nodes (0.1–1.4%). However, higher frequencies of CD3^+^NKp46^+^ cells were found in non-lymphatic organs like liver (0.6–2.8%) and lung (0.9–10.6%). This might indicate that this lymphocyte population plays a role in immune surveillance in these organs.

### CD3^+^NKp46^+^ Lymphocytes Show a Mixed NK-/T-Cell Phenotype

**Figure 2:**
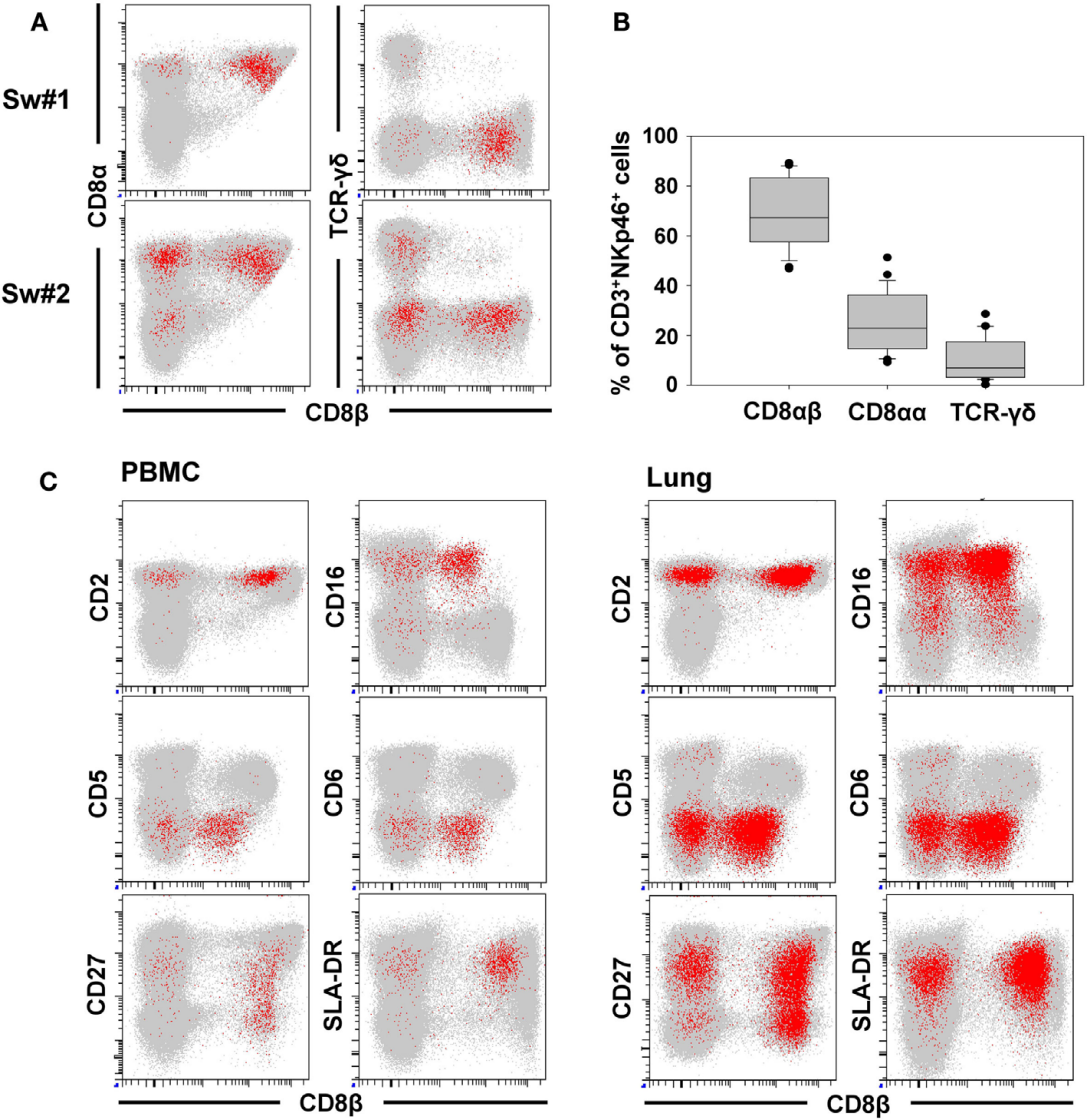
**Phenotype of porcine CD3^+^NKp46^+^ lymphocytes in blood and lung**. CD3^+^NKp46^+^ lymphocytes derived from blood and lung were analyzed for their expression of different NK- and T-cell-associated surface markers by multicolor FCM. CD3^+^NKp46^+^ cells are indicated in red, total live lymphocytes are shown in light gray as background. **(A)** Coexpression of CD8α/CD8β/TCR-γδ on CD3^+^NKp46^+^ blood lymphocytes for two representative animals. **(B)** Box-plots show the frequencies of CD8α^+^/CD8β^+^/TCR-γδ^+^ cells within CD3^+^NKp46^+^ lymphocytes of 24 animals. **(C)** Expression of CD2/CD5/CD6/CD16/CD27/SLA-DR and CD8β in CD3^+^NKp46^+^ cells derived from blood (left) and lung (right) for one individual animal. Data are representative for analyses of six different individuals.

For further phenotypic characterization of CD3^+^NKp46^+^ lymphocytes, the expression of T-cell-associated markers was analyzed in blood of individual animals by FCM (Figure 2, CD3^+^NKp46^+^ cells shown in red). In most animals, a coexpression of CD8α and CD8β could be observed on the majority of CD3^+^NKp46^+^ cells (Figures 2A,B). Nevertheless, CD8β^−^ cells could also be identified to varying degrees with a frequency of up to 50% of CD3^+^NKp46^+^ lymphocytes in individual animals (Figure 2A, animal Sw#2). Partially, this CD8β^−^ phenotype was associated with coexpression of the TCR-γδ (Figures 2A,B). Overall results from 24 animals (Figure 2B) showed that the vast majority of CD3^+^NKp46^+^ lymphocytes coexpressed the CD8αβ heterodimer, therefore, showing phenotypic accordance with porcine cytotoxic T lymphocytes. About 10% of the CD3^+^NKp46^+^ cells expressed the TCR-γδ, which coincided with a CD8αα homodimer expression. No obvious expression of CD4 could be observed within the CD3^+^NKp46^+^ cells in blood of all animals (data not shown).

In the next step, CD3^+^NKp46^+^ lymphocytes were analyzed for further markers associated with either NK cells or T cells (Figure 2C). Therefore, lymphocytes derived from blood (Figure 2C, left side), lymph node, and spleen (data not shown), as well as lung (Figure 2C, right side) were analyzed by FCM. CD2, that is expressed on NK cells and T cells were present on all CD3^+^NKp46^+^ lymphocytes. The vast majority of CD3^+^NKp46^+^ cells were negative for the T-cell-related markers CD5 and CD6. However, most cells expressed CD16, which is highly associated with a NK-cell phenotype in the pig (36). CD27 and SLA-DR, both expressed on subpopulations of porcine NK and T cells (30, 35), likewise were present to various degrees on CD3^+^NKp46^+^ cells. Comparable results for all analyzed surface markers could also be observed for lymph node and spleen (data not shown). Additionally, CD8β was included in the staining panel to investigate potential correlations in regard to the CD8β^−/+^ subsets within CD3^+^NKp46^+^ lymphocytes. No obvious differences for most cell surface markers analyzed could be observed within the CD8β^−^ and CD8β^+^ subsets. Nevertheless, the majority of CD8β^−^ CD3^+^NKp46^+^ cells expressed CD27, while a more heterogeneous CD27 expression pattern was observed within the CD8β^+^ subset (Figure 2C, bottom row).

**Figure 3:**
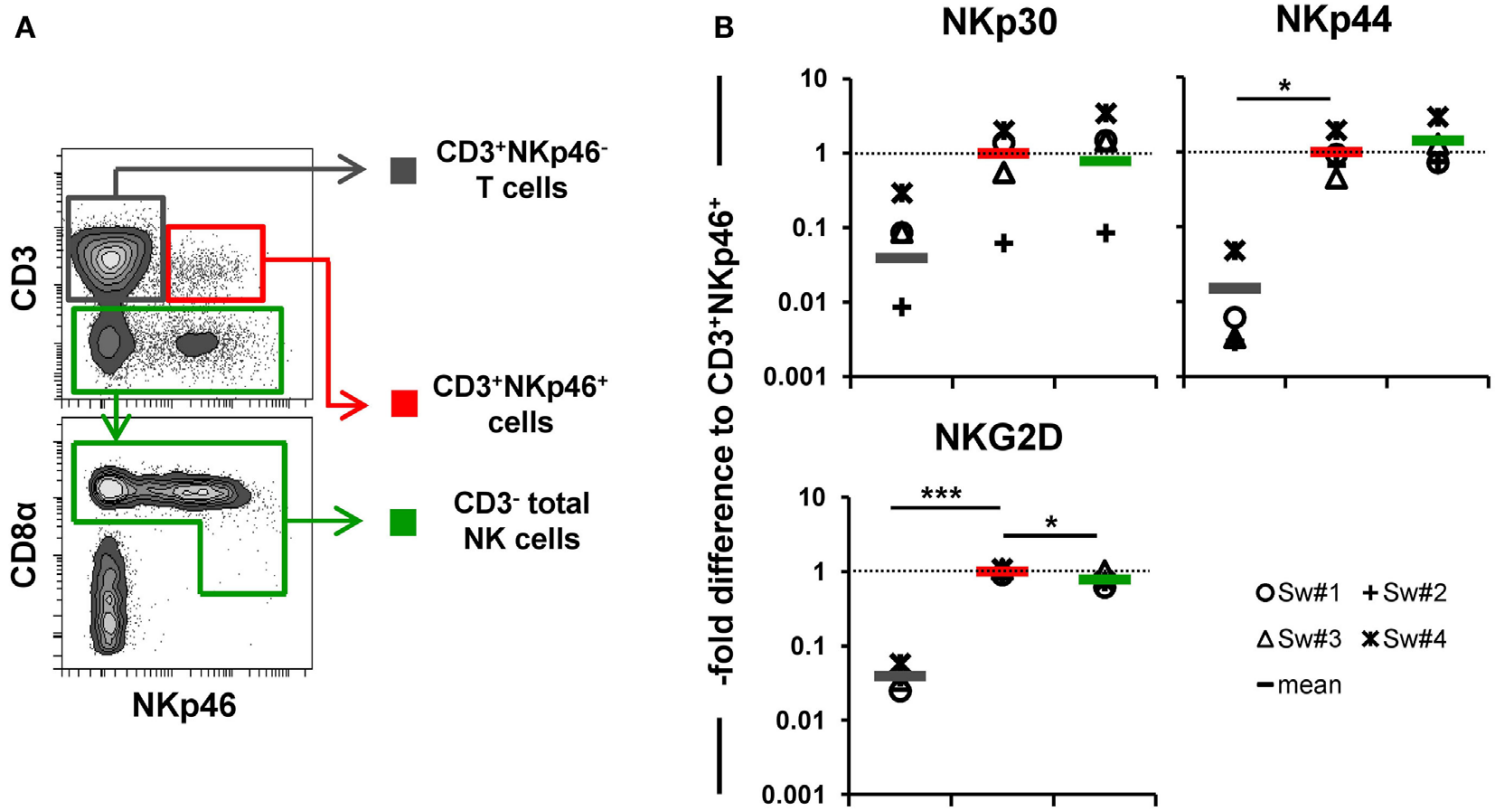
**Expression of NK-associated receptor mRNAs in CD3^+^NKp46^+^ lymphocytes**. **(A)** PBMC were FACS sorted into CD3^+^NKp46^−^ T cells (gray), CD3^+^NKp46^+^ cells (red), and total CD3^−^ NK cells (green). **(B)** Sorted lymphocyte subsets were analyzed for their mRNA expression levels of NKp30, NKp44, and NKG2D by quantitative RT-PCR. The 2^−ΔΔCq^ values for the target genes of each individual animal (*n* = 4) are shown as fold differences relative to the mean of CD3^+^NKp46^+^ cells from all animals, which was set to a value of 1. Colored lines represent mean values of the respective lymphocyte subsets. Significant differences between the CD3^+^NKp46^+^ cells and T as well as NK cells are indicated (*p ≤ 0.05, ***p ≤ 0.001).

In addition to NKp46, we further wanted to investigate whether other NK-associated activating receptors like NKG2D as well as NKp30 and NKp44 were expressed on the CD3^+^NKp46^+^ lymphocytes. Due to the lack of species-specific mAbs for these markers, FACS-sorted lymphocyte subsets were analyzed by quantitative RT-PCR (Figure 3). PBMC of four animals were FACS-sorted in the CD3^+^NKp46^+^ subset (Figure 3A, shown in red) and compared with either CD3^+^NKp46^−^ T cells (shown in gray) or CD3^−^ total NK cells (shown in green). For NKp30, NKp44, as well as NKG2D similar mRNA expression levels between CD3^+^NKp46^+^ lymphocytes and total NK cells were found, whereas reduced expression levels were observed for T cells (Figure 3B).

Therefore, phenotypic analysis indicated a mixed phenotype of CD3^+^NKp46^+^ lymphocytes, comprising both NK- and T-cell-associated molecules.

### CD3^+^NKp46^+^ Lymphocytes Respond to Cytokine Stimulation in a NK-Like Fashion

**Figure 4:**
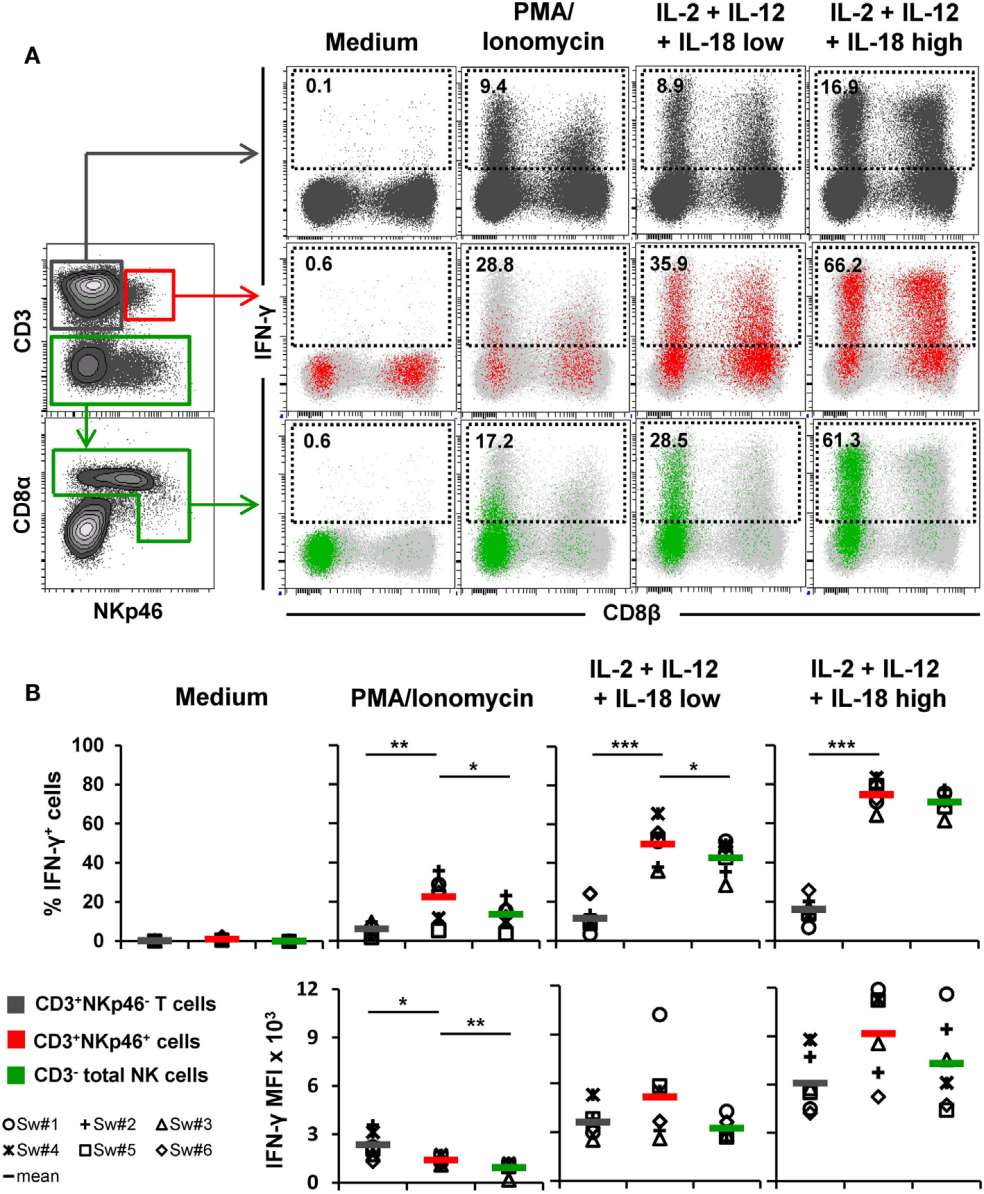
**IFN-γ production of CD3^+^NKp46^+^ lymphocytes**. PBMC were stimulated either with PMA/Ionomycin for 4 h or with a combination of rpIL-2 + rpIL-12 + rpIL-18 for 24 h. IL-18 was used at two different concentrations (low: 5 ng/ml, high: 25 ng/ml). Cells cultured in medium alone served as negative control. Following stimulation, intracellular IFN-γ expression of CD3^+^NKp46^+^ lymphocytes (red) was analyzed and compared with CD3^+^NKp46^−^ T cells (gray) and total CD3^−^ NK cells (green). **(A)** IFN-γ expression of the respective lymphocyte subsets is shown in the dot-plots on the right in combination with CD8β for one representative animal. Analyzed cell subsets are highlighted in color, total lymphocyte population is shown in light gray in the background. Percentages of IFN-γ^+^ cells within respective subsets are indicated. **(B)** Frequencies of IFN-γ^+^ cells (upper row) as well as median fluorescence intensity of IFN-γ^+^ cells (bottom row) within the three analyzed lymphocyte subsets in blood of six animals are shown. Mean values are represented by colored bars. Significant differences between the CD3^+^NKp46^+^ cells and T as well as NK cells are indicated (*p ≤ 0.05, **p ≤ 0.01, ***p ≤ 0.001).

**Figure 5:**
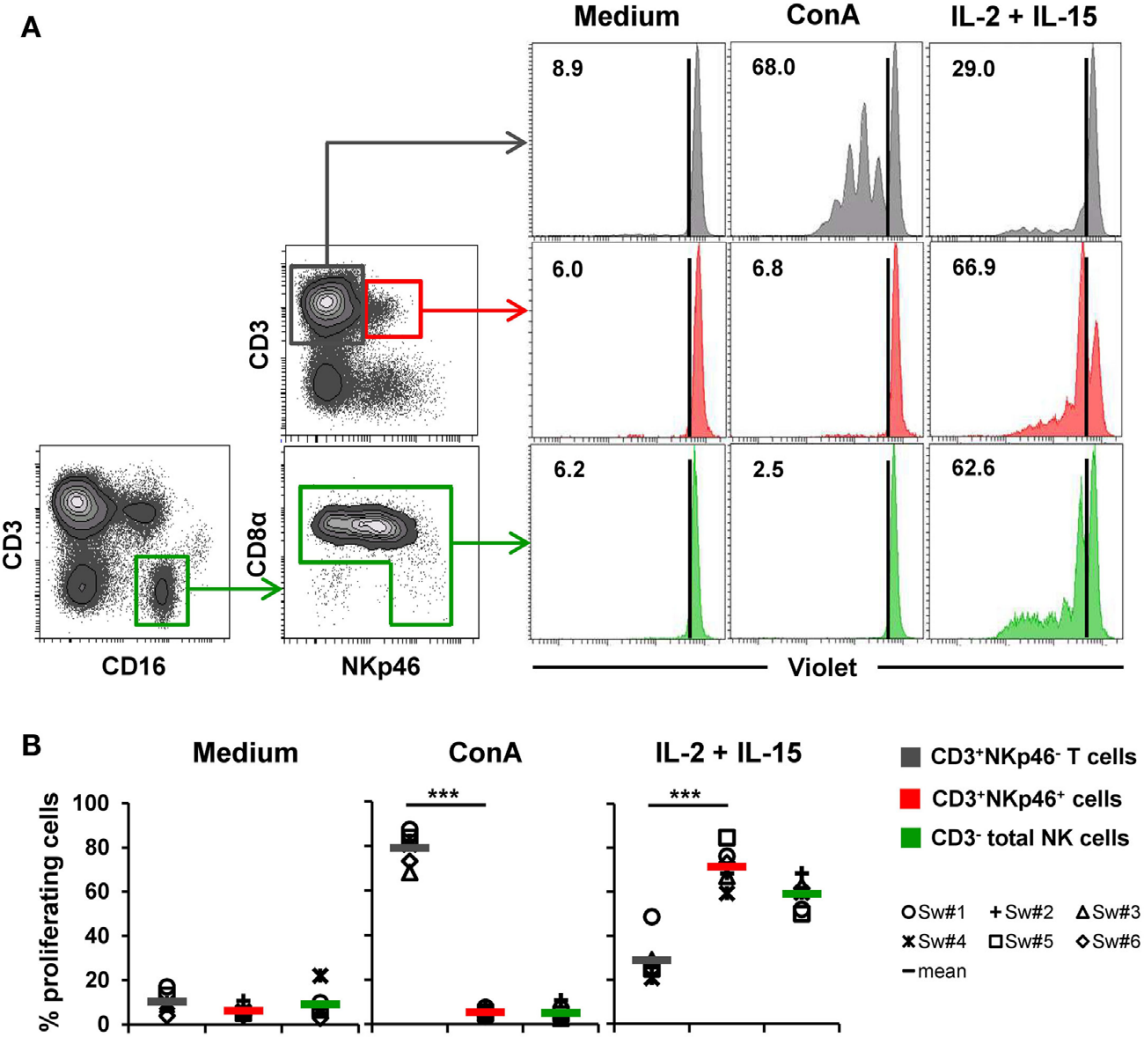
**Proliferative capacity of CD3^+^NKp46^+^ lymphocytes**. PBMC were stained with Violet Cell Trace Dye to analyze proliferating cells following stimulation with either ConA or rpIL-2 and rpIL-15 for 4 days. Cells cultured in medium alone served as negative control. Proliferation of CD3^+^NKp46^+^ lymphocytes (red) was compared with CD3^+^NKp46^−^ T cells (gray) and total CD3^−^ NK cells (green). For NK cells, an additional pre-gating on CD3^−^CD16^+^ was performed. **(A)** Histograms show proliferation of the different lymphocyte subsets following respective stimulation for one representative animal. Percentages of proliferating cells within respective subsets are indicated. **(B)** Frequencies of proliferating cells within the three analyzed lymphocyte subsets in blood of six animals are shown. Mean values are represented by colored bars. Significant differences between the CD3^+^NKp46^+^ cells and T as well as NK cells are indicated (***p ≤ 0.001).

In a next series of experiments, we wanted to address whether CD3^+^NKp46^+^ lymphocytes share functional properties with either NK cells or T cells. Therefore, IFN-γ production (Figure 4) and proliferative capacity (Figure 5) of blood-derived CD3^+^NKp46^+^ cells (shown in red) were investigated and compared with CD3^+^NKp46^−^ T cells (shown in gray) and CD3^−^ total NK cells (shown in green).

For detection of intracellular IFN-γ production (Figure 4), PBMC of six animals were stimulated either for 4 h with PMA/Ionomycin or a combination of rpIL-2, rpIL-12, and rpIL-18 for 24 h. This cytokine combination effectively induces IFN-γ production in porcine NK cells, as shown in previous studies (15, 16, 31). Cells cultured in medium alone did not show any cytokine production (Figures 4A,B, “Medium”). PMA/Ionomycin stimulation induced moderate frequencies of IFN-γ producing cells in all three lymphocyte subsets (Figures 4A,B, “PMA/Ionomycin”). Although the average number of IFN-γ^+^ cells was lower in T cells (6.2 ± 3.2%) compared with CD3^+^NKp46^+^ (22.5 ± 11.6%) and NK cells (13.6 ± 6.7%, Figure 4B), T cells showed at least twice as much IFN-γ production on a per-cell level after PMA/Ionomycin stimulation, indicated by the increased MFI (Figure 4B). As expected, cytokine stimulation induced high frequencies of IFN-γ-producing NK cells (42.7 ± 9.1%, Figures 4A,B, “IL-2 + IL-12 + IL-18 low”), which were even more increased with higher levels of rpIL-18 (71.0 ± 5.7%, Figures 4A,B, “IL-2 + IL-12 + IL-18 high”). Comparable frequencies could be observed for CD3^+^NKp46^+^ lymphocytes with an average number of 49.8 ± 11.2% (IL-18 low) and 74.8 ± 6.9% (IL-18 high) IFN-γ^+^ cells. Additionally, IFN-γ production on a per-cell level could be enhanced with higher levels of IL-18 in both subsets (Figure 4B). Although IFN-γ^+^ T cells also slightly increased with higher concentrations of rpIL-18 (IL-18 low: 11.5 ± 7.0%; IL-18 high: 16.1 ± 6.5%), the average number of IFN-γ^+^ T cells was several-fold lower than in the other two lymphocyte subsets. Expression of CD8β was investigated along with IFN-γ production to address potential functional differences in regard to the CD8β^−/+^ phenotypes of CD3^+^NKp46^+^ cells. However, no obvious CD8β-correlated differences for IFN-γ^+^ subsets could be observed in all animals tested (Figure 4A and data not shown).

To assess proliferation, Cell Trace Violet-labeled PBMC of six animals were stimulated for 4 days with either ConA or a combination of rpIL-2 and rpIL-15 (Figure 5). Since CD3-downregulation on T cells can be observed after prolonged *in vitro* stimulation (30), the gating strategy for NK cells in this setup was further extended by gating on CD3^−^CD16^+^ cells to exclude “CD3 false-negative” cells in the CD3^−^ gate (Figure 5A). As expected, T cells vigorously proliferated following ConA stimulation (79.2 ± 7.2%, Figure 5A,B, “ConA”). In contrast, minor proliferation rates after mitogen stimulation were observed for CD3^+^NKp46^+^ cells (5.3 ± 1.8%) and NK cells (5.0 ± 3.2%). After stimulation with rpIL-2 and rpIL-15, CD3^+^NKp46^+^ cells showed high proliferation rates (71.4 ± 8.7%, Figure 5A,B, “IL-2 + IL-15”) that slightly exceeded those of NK cells (59.0 ± 6.9%), whereas T cells only showed a moderate response to cytokine stimulation (28.9 ± 10.0%). Unstimulated cells showed minor proliferation rates in all observed lymphocyte populations (Figures 5A,B, “Medium”). Similar to IFN-γ production, we could not observe any CD8β-correlated differences for proliferation within the CD3^+^NKp46^+^ population (data not shown).

Taken together, CD3^+^NKp46^+^ lymphocytes showed proliferation rates and IFN-γ production similar to NK cells following cytokine stimulation.

### CD3^+^NKp46^+^ Lymphocytes Display Cytolytic Properties Comparable to NK Cells

One of the major properties of NK cells is their ability to spontaneously kill susceptible target cells, mediated by effector molecules like perforin. Since the functional properties of CD3^+^NKp46^+^ lymphocytes investigated so far were rather comparable to NK cells, we investigated if they likewise show these properties.

**Figure 6:**
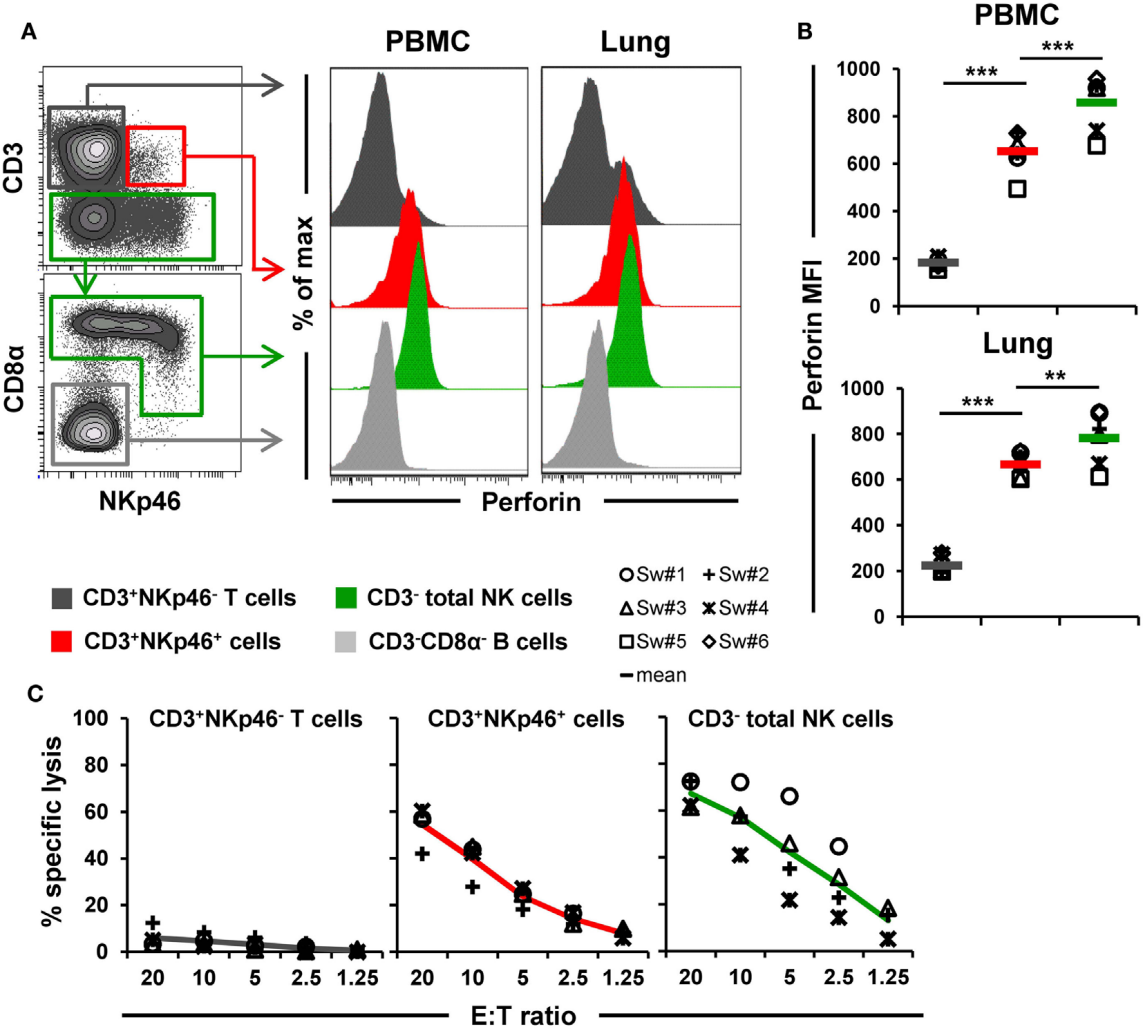
**Cytolytic properties of CD3^+^NKp46^+^ lymphocytes**. **(A)** Lymphocytes derived from blood and lung were analyzed for perforin expression. Histograms show the perforin expression levels of CD3^+^NKp46^+^ cells (red) compared with CD3^+^NKp46^−^ T cells (gray) and total CD3^−^ NK cells (green) for one representative animal. Results for CD3^−^CD8α^−^ cells, mainly consisting of B cells, are shown in light gray and serve as negative reference. **(B)** Median fluorescence intensities of perforin within respective lymphocyte subsets are shown for PBMC and lung for six animals. Mean values are represented by colored bars. Significant differences between the CD3^+^NKp46^+^ cells and T as well as NK cells are indicated (**p ≤ 0.01, ***p ≤ 0.001). **(C)** FACS-sorted CD3^+^NKp46^−^ T cells (gray), CD3^+^NKp46^+^ cells (red), and total CD3^−^ NK cells (green) from blood were stimulated with rpIL-2 and rpIL-15 for 36 h and subsequently used in a 4h-cytotoxic assay with K562 as target cell line. Respective lymphocyte subsets were tested at five different E:T ratios: 20:1, 10:1, 5:1, 2.5:1, and 1.25:1. Results obtained from analyses of four animals are shown. Colored lines represent mean values of the respective lymphocyte subsets.

*Ex vivo* perforin expression was analyzed by FCM in lymphocytes isolated from blood and lungs of six animals (Figures 6A,B). As a negative reference, CD3^−^CD8α^−^ cells, mainly consisting of B cells, were analyzed in parallel (Figure 6A, shown in light gray). CD3^+^NKp46^+^ cells derived from blood and lung showed perforin expression levels (MFI of 652 ± 88 for blood and 667 ± 54 for lung) that were close to expression levels of NK cells (average MFI of 857 ± 118 for blood and 782 ± 117 for lung, respectively). Total T cells on the other hand only showed minor perforin expression in both organs.

Additionally, the ability of CD3^+^NKp46^+^ lymphocytes to kill the human leukemia cell line K562 was investigated (Figure 6C). Therefore, PBMC of four animals were FACS-sorted in CD3^+^NKp46^−^ T cells, CD3^−^ total NK cells, and the CD3^+^NKp46^+^ subset and tested in a 4-h cytotoxic assay after stimulation with rpIL-2 and rpIL-15 for 36 h. As expected, T cells showed hardly any cytolytic activity toward the K562 cell line at any E:T ratio. In contrast, NK cells efficiently lysed the target cells, reaching specific lysis of 67.3 ± 6.1% at the highest E:T ratio of 20:1. Likewise, CD3^+^NKp46^+^ lymphocytes showed spontaneous lytic activity in an E:T-ratio dependent manner with only slightly reduced levels compared with total NK cells (average specific lysis of 54.5 ± 8.5% at an E:T ratio of 20:1). These data suggest that CD3^+^NKp46^+^ lymphocytes are capable of spontaneous lytic activity, comparable to NK cells.

### Cross-Linking of NKp46 and CD3 Leads to Degranulation of CD3^+^NKp46^+^ Lymphocytes

**Figure 7:**
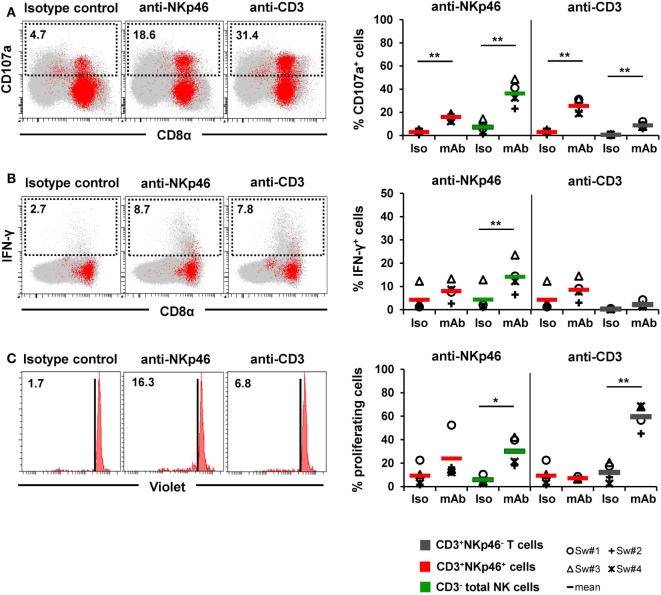
**Activation of CD3^+^NKp46^+^ lymphocytes by receptor cross-linking**. Receptor-mediated degranulation, IFN-γ production, and proliferation was assessed by multicolor FCM in response to cross-linking of NKp46 or CD3 by plate-bound mAbs. Isotype-matched irrelevant antibodies served as control. Activation of CD3^+^NKp46^+^ lymphocytes (red) derived from blood of four different individuals was analyzed and compared with either total CD3^−^ NK cells (green) or CD3^+^NKp46^−^ T cells (gray). Gating for FCM analyses was performed as displayed in the previous figures. **(A)** PBMC were pre-activated with rpIL-2 and rpIL-15 for 24 h and receptor-mediated degranulation was assessed by CD107a expression on the cell surface after 4 h incubation with plate-bound mAbs. **(B)** PBMC were pre-activated with rpIL-2 and rpIL-18 for 24 h and intracellular IFN-γ production was measured after receptor cross-linking for 4 h. **(C)** Total PBMC were stained with Violet Cell Trace Dye and cultured for 4 days in the presence of plate-bound mAbs. **(A–C)** Dot-plots and histograms show results of CD3^+^NKp46^+^ lymphocytes for one representative animal. CD3^+^NKp46^+^ cells are shown in red, total lymphocytes are shown in light gray as background. Frequencies of CD107a^+^ and IFN-γ^+^ as well as proliferating cells within lymphocyte subsets are shown for analyses of four animals in the graphs. Mean values are represented by colored bars. Significant differences between stimulated and non-stimulated CD3^+^NKp46^+^ cells, T and NK cells are indicated (*p ≤ 0.05, **p ≤ 0.01).

Since the data so far indicated that CD3^+^NKp46^+^ cells functionally resemble NK cells, we further addressed the functionality of NKp46 and CD3 molecules on this lymphocyte subset. For this purpose, PBMC of four animals were stimulated with plate-bound antibodies against either NKp46 or CD3 to analyze activation of the CD3^+^NKp46^+^ cells (Figure 7).

Triggering of NKp46 was already shown to induce cytolytic activity in porcine NK cells (15, 16). Therefore, in a first attempt, degranulation of CD3^+^NKp46^+^ cells (shown in red) was analyzed in a CD107a assay by FCM (Figure 7A) after NKp46 cross-linking. Total NK cells (shown in green) were analyzed in parallel. Ligation of NKp46 resulted in degranulation of NK cells, shown by a fivefold increase in CD107a^+^ cells (36 ± 11%) compared with isotype-matched control antibodies. Likewise, triggering of NKp46 on CD3^+^NKp46^+^ lymphocytes led to an increase in the frequency of CD107a^+^ cells (16 ± 3% compared with 3 ± 2%). Triggering of CD3 also induced degranulation in CD3^+^NKp46^+^ cells (26 ± 7% CD107a^+^ cells). An increase in CD107a^+^ cells could also be observed within CD3-stimulated total T cells (shown in gray). These results indicate that NKp46 and the CD3/TCR complex are functional in CD3^+^NKp46^+^ lymphocytes.

In addition to cytolytic activity, NKp46 cross-linking is known to induce IFN-γ production in NK cells. Nevertheless, experiments with porcine PBMC showed that although IFN-γ^+^ cells could be detected in NK cells after cross-linking of NKp46 (14 ± 7%, Figure 7B), no obvious IFN-γ production could be observed within CD3^+^NKp46^+^ lymphocytes compared with isotype-matched control antibodies (Figure 7B). Similarly, no IFN-γ production could be observed in CD3^+^NKp46^+^ lymphocytes as well as CD3^+^NKp46^−^ T cells after CD3 cross-linking. Furthermore, cross-linking of CD3 did not induce proliferation in CD3^+^NKp46^+^ cells in contrast to T cells where about half of the cells proliferated (60 ± 11%, Figure 7C). Interestingly, proliferation could be detected in total NK cells after NKp46 cross-linking (30 ± 12%), but no such effect was observed for the CD3^+^NKp46^+^ subset.

### CD3^+^NKp46^+^ Lymphocytes Are Present in Increased Frequencies in the Lungs of Influenza-Infected Animals

**Figure 8:**
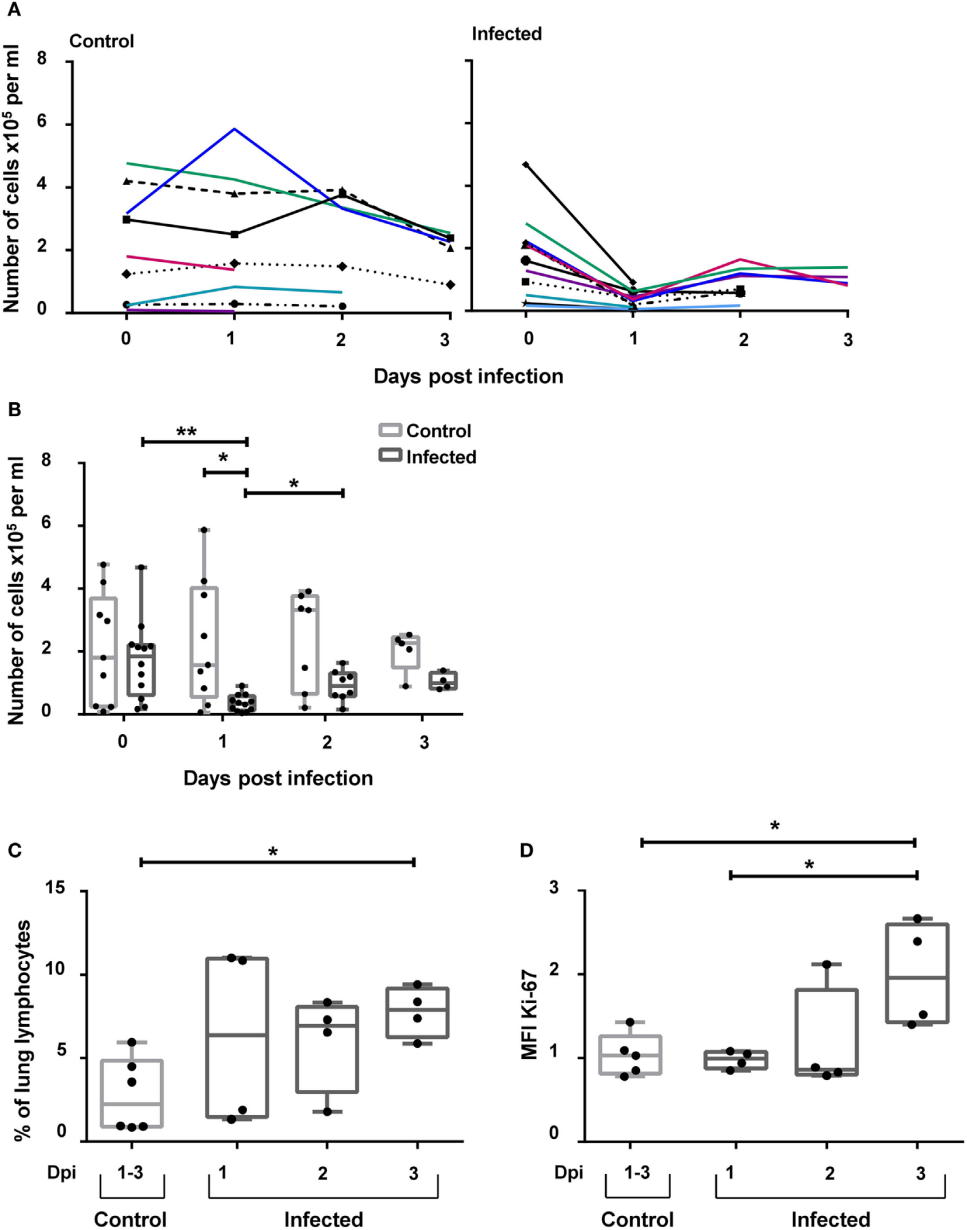
**CD3^+^NKp46^+^ lymphocytes in influenza A-infected piglets**. CD3^+^NKp46^+^ lymphocytes from blood and lung of influenza A-infected piglets and healthy control animals were analyzed by flow cytometry on days 1–3 after infection. **(A)** Absolute numbers of CD3^+^NKp46^+^ cells in PBMC of controls (left, *n* = 9) and infected animals (right, *n* = 12) are shown in the course of influenza infection. **(B)** Box-plots show absolute numbers of CD3^+^NKp46^+^ cells in PBMC on individual days post-infection. **(C)** Frequencies of CD3^+^NKp46^+^ cells among lung lymphocytes in control (*n* = 6) and infected animals (*n* = 12) in the course of influenza infection. **(D)** Box-plots show median fluorescence intensity of Ki-67 in CD3^+^NKp46^+^ lung cells. Significant differences between infected and control animals as well as distinct study days are indicated (*p ≤ 0.05, **p ≤ 0.01).

Recently, it was demonstrated that NKp46^+^ lymphocytes accumulate in the vicinity of influenza A-infected cells in the lung of infected pigs (17). To investigate the possibility that besides NKp46^+^ NK cells, CD3^+^NKp46^+^ cells might also be involved in this process, this lymphocyte population was analyzed in animals experimentally infected with the 2009 pandemic H1N1 influenza virus strain. Frequencies of CD3^+^NKp46^+^ lymphocytes were analyzed in blood and lung at days 1–3 p.i. and compared with non-infected control animals. A significant decrease in the total number of CD3^+^NKp46^+^ lymphocytes in PBMC could be observed in infected animals 1 day p.i. compared with control animals (Figures 8A,B). Furthermore, a significant increase in the frequency of CD3^+^NKp46^+^ lymphocytes could be detected in the lungs of infected animals compared with the control group on day 3 p.i. (Figure 8C). To investigate proliferative activity of CD3^+^NKp46^+^ cells, expression of Ki-67 was analyzed. Compared with analyses from day 1 p.i., a significant increase in the MFI of Ki-67 could be detected within CD3^+^NKp46^+^ cells in the lung on day 3 p.i. (Figure 8D). The MFI was also significantly higher compared with Ki-67 expression in CD3^+^NKp46^+^ cells of the control group.

## Discussion

Expression of the activating receptor NKp46 in combination with lack of a TCR/CD3 complex is commonly used to describe NK cells in most mammalian species (9). We could identify a lymphocyte subset in pig with surface expression of both NKp46 and CD3, thereby displaying a mixed NK-/T-cell phenotype.

Likewise, rare populations of CD3^+^NKp46^+^ lymphocytes have been identified in other species (9, 18–22, 24, 25, 37) and have been either assigned to TCR-αβ^+^ or TCR-γδ^+^ T cells. These NKp46^+^ T-cell subsets show very heterogeneous phenotypes and seem to be derived from different progenitors rather than representing one distinct lymphocyte population. In most cases, they are described as T cells acquiring NK-cell properties upon specific stimuli. This was mainly reported to be a result of chronic activation of the TCR (19), chronic infections, and inflammatory conditions (21, 22, 37), as well as prolonged stimulation with IL-15 (23, 38). Additionally, genetic reprograming of T cells into NKp46^+^ cells after transcription factor deletion has been described in mice (39). However, CD3^+^NKp46^+^ lymphocyte subsets that are present in apparently healthy individuals have been reported in human, mouse, cattle, and dog (9, 20, 24, 25), although at very low frequencies. The porcine CD3^+^NKp46^+^ cells described in this study were also identified in clinically healthy animals but this does not exclude the induction of these cells in certain physiological or pathologic conditions. Indeed, we could show that stimulation with IL-2 and IL-15 led to *in vitro* proliferation of this cell population. Also, in influenza-infected animals an increase of Ki-67 expression in lung-derived CD3^+^NKp46^+^ cells was observed.

Besides the expression of CD3, the majority of porcine CD3^+^NKp46^+^ lymphocytes expressed the CD8αβ heterodimer and, therefore, displayed phenotypic characteristics of porcine cytolytic T cells (34, 35). Additionally, a minor population of CD8β^−^ CD3^+^NKp46^+^ cells could be identified that belongs to TCR-γδ lymphocytes. Yet, porcine CD3^+^NKp46^+^ cells did not express CD5 and CD6, markers that are normally associated with a T-cell phenotype in the pig (35, 40, 41). Instead, other NK-associated markers were expressed by CD3^+^NKp46^+^ lymphocytes at comparable levels to porcine NK cells, like the activating receptors CD16 and NKG2D and the NCR family members NKp30 and NKp44. Likewise, the effector molecule perforin was found to be expressed in all CD3^+^NKp46^+^ cells. Thus, indicating that these cells have more phenotypic similarities with porcine NK cells. Earlier studies in pigs describe a CD3^+^ lymphocyte population that also expressed CD16 and perforin (36). The majority of these cells were CD5^−^CD6^−^ and were suggested to represent porcine NKT cells. The CD3^+^NKp46^+^ cells, thus, might also belong into this category of non-conventional T cells. In a more recent publication, a small porcine lymphocyte subset reacting with α-galactosylceramide-loaded CD1d tetramers was described and defined as porcine invariant NKT (iNKT) cells (42). As these cells were described to have a CD5^high^CD16^low/−^ phenotype, porcine CD3^+^NKp46^+^ cells might rather belong to non-CD1d-restricted NKT-like cells.

Despite their mixed NK-/T-cell phenotype, functional properties of the porcine CD3^+^NKp46^+^ lymphocytes resemble those of NK cells. Whereas no proliferation could be detected after ConA stimulation, CD3^+^NKp46^+^ cells responded in a NK-like manner to cytokine stimulation with proliferation and increased IFN-γ production. Moreover, porcine CD3^+^NKp46^+^ lymphocytes showed spontaneous killing activity against the NK-susceptible target cell line K562, comparable to porcine NK cells. As it was already demonstrated that killing of K562 cells by porcine NK cells is NKp46-independent (15), CD3^+^NKp46^+^ lymphocytes must harbor other mechanisms for recognition and killing of xenogeneic target cells. Comparable to our results, most of the CD3^+^NKp46^+^ subsets identified in mouse (19, 20) and human (20, 22) produced IFN-γ in response to cytokines or triggering of NKp46 receptor. This ability might be closely linked to the expression of the activating receptor itself, as it was shown that induction of NKp46 expression during NK-cell maturation was associated with a higher ability to secrete IFN-γ in response to IL-12 and IL-18 (10). Interestingly, in cattle, only an *in vitro*-generated TCRγδ^+^NKp46^+^ lymphocyte subset was capable of producing IFN-γ in response to cytokines (23), whereas the CD3^+^NKp46^+^ lymphocytes found *ex vivo* did not respond to cytokine stimulation or receptor triggering (24). Additionally, we could show that porcine CD3^+^NKp46^+^ cells have a functional NKp46 signaling pathway, as triggering of the receptor led to CD107a upregulation. Likewise, cytolytic function of CD3^+^NKp46^+^ cells after receptor activation could be found in human NKp46^+^ CTL (22) and the bovine CD3^+^NKp46^+^ non-conventional T cells (24).

Although functional NK-cell characteristics dominate in porcine CD3^+^NKp46^+^ lymphocytes, we could also demonstrate that CD3 stimulation by plate-bound mAbs leads to degranulation of CD3^+^NKp46^+^ cells. This indicates a functional TCR complex. Up to date, analyses on TCR-αβ expression are hampered by the lack of anti-porcine TCR-αβ-specific mAbs. Nonetheless, surface expression of the CD3ε chain is strongly connected to TCR coexpression as assembly with the TCR chains is essential for transport of CD3ε to the cell surface (43, 44). Therefore, identification of the CD3^+^NKp46^+^ lymphocytes using mAbs specific for the extracellular part of the porcine CD3ε chain (45, 46) suggests expression of TCR complexes on these cells. This assumption is further supported by the identification of a small TCR-γδ-expressing fraction of porcine CD3^+^NKp46^+^ lymphocytes. NK cells on the other hand do not express CD3 proteins with the exception of CD3ζ that serves as a signaling adaptor molecule (47). Rare CD3ε transcripts and proteins could, so far, only be detected in human fetal NK cells and in *in vitro*-generated NK-cell clones, but are apparently restricted to the cytoplasm (48, 49).

Nevertheless, analyses of the TCR repertoire would be relevant for a further characterization of porcine CD3^+^NKp46^+^ cells, an issue we aim to address in the future. So far, a highly diverse TCR repertoire was found in bovine CD3^+^NKp46^+^ cells (24), whereas a rather restricted TCR repertoire was found in the human NKp46^+^ CTL (22) as it was described for the semi-conserved TCR repertoires in most non-conventional T cells like iNKT and mucosal-associated invariant T (MAIT) cells (50).

At the moment, it remains an open question if porcine CD3^+^NKp46^+^ lymphocytes can be activated in an antigen-specific manner *via* their TCR. Nevertheless, due to their NK-like phenotype and function, a potential role in the early defense against infections can be assumed. The role of NKp46-expressing NK cells in the course of influenza infections could already be shown in several studies. NKp46 can bind to hemagglutinin of influenza virus (5, 7) and seem to be actively involved in the defense against infection. Activation of NKp46 leads to killing of infected cells (7, 51) and absence of the receptor in mice results in a lethal outcome of the disease (52). Moreover, NKp46^+^ NK cells can be found in higher frequencies in lungs of infected mice (53). Likewise, in pig, it has recently been shown that NKp46^+^ cells seem to be involved in the immune response against influenza. NKp46^+^ lymphocytes accumulate in influenza virus-infected areas of the lung, and in *in vitro* experiments, influenza HA is recognized by porcine NKp46 (17). Comparable to the NKp46^high^ NK-cell subset in the pig (15, 17), also the porcine CD3^+^NKp46^+^ lymphocytes seem to preferentially reside in non-lymphatic organs like the lung, indicating a role of these cells in the immune surveillance of this organ. Indeed, we could demonstrate a decrease of CD3^+^NKp46^+^ cells in blood in the early course of influenza infection, which is in line with results obtained with CD3^−^NKp46^+^ NK cells (17). This decrease was accompanied by an increase of CD3^+^NKp46^+^ cells in lungs of infected animals. Additionally, proliferation of these lung-derived cells could be shown by their elevated levels of Ki-67 expression. Interestingly, this increase in Ki-67 expression could only be detected in the CD3^+^NKp46^+^ cells and not in CD3^−^NKp46^+^ NK cells (17). Also, in cattle, a potential role of the CD3^+^NKp46^+^ lymphocyte subset as an effector population has been described as they expand during infection with the parasitic protozoan *Theileria parva* and show cytolytic activity toward *Theileria parva*-infected cells (24).

In conclusion, we could identify a CD3^+^NKp46^+^ lymphocyte subset in swine that shows a mixed NK-/T-cell phenotype with dominating NK-cell properties. Nonetheless, besides NK-signaling pathways, our data also suggest a functional CD3/TCR receptor complex, which may allow responsiveness to a broader range of pathogens. Lymphocytes with these combined properties may also be well suited to fill a gap between innate and adaptive immune responses during infections. Such cells might be of relevance in infections where both an evasion of the innate response by NK cells and subsequent delay of adaptive responses occur, as described for infections of pigs with the porcine reproductive and respiratory syndrome virus (PRRSV) (54). Although an antigen-specific immune response of these newly characterized cells has yet to be verified, induced proliferation during influenza infection suggests that this lymphocyte subset is capable of antigen-specific clonal expansion. As there is evidence that also innate cells, like NK cells, can exhibit memory functions (55–57), it is reasonable to speculate that the same accounts for the NK-/T-cell CD3^+^NKp46^+^ lymphocytes. This would mark them as attractive targets in vaccine design. As porcine CD3^+^NKp46^+^ cells are already enriched in the lungs of pigs kept in ordinary environmental conditions, we propose that these lymphocytes are important players in the immune responses against frequently encountered respiratory infections of pigs, like influenza A virus and PRRSV.

## Author Contributions

KM, AS, and WG were responsible for conception and design of the study. KM performed experiments, analyzed data, and wrote the manuscript. MS and ST performed laboratory work and experiments. HF and AKS were responsible for the influenza infection study, including experiments and data analyses. AM, CD, and SH performed qPCR design, experiments, and data analyses. WG and AS interpreted data and supervised the study. All authors read and approved the final manuscript.

## Conflict of Interest Statement

The authors declare that the research was conducted in the absence of any commercial or financial relationships that could be construed as a potential conflict of interest.
