# Supplementary material for: Porcine CD3+NKp46+ Lymphocytes Have NK-Cell Characteristics and Are Present in Increased Frequencies in the Lungs of Influenza-Infected Animals
Source: Front Immunol. 2016 Jul 14;7:263. doi: 10.3389/fimmu.2016.00263 (PMC4943943; doi:10.3389/fimmu.2016.00263)
Supplement: Supplementary file 1 [file Data_Sheet_1.PDF]

Supplementary Material

Porcine $CD3^+NKp46^+$ Lymphocytes Have NK-Cell Characteristics and Are Present in Increased Frequencies in the Lungs of Influenza-Infected Animals

Kerstin H. Mair*, Maria Stadler, Stephanie C. Talker, Hilde Forberg, Anne K. Storset, Andrea Müllebner, J. Catharina Duvigneau, Sabine E. Hammer, Armin Saalmüller and Wilhelm Gerner

***Corresponding author:** Kerstin H. Mair, Kerstin.Mair@vetmeduni.ac.at

Supplementary Data: Optimisation and validation of qPCR assays for target gene-specific primers in the pig.

Information on Intron-spanning primers.

Primers or products spanning Exon-Exon junctions are indicated in the table including length of intron in base pairs (bp).

Target	Exon junctions in	Intron size (bp)
**NKp30**	product	1537
**NKp44**	product	5025

Optimised protocol for the amplification of target sequences by qPCR.

Target	Annealing/Extension temp (°C)/time (sec)	ΔCt (RT+ to RT-)	Slope	Correlation coefficient (Pearson) R^2	Verified dynamic range	Product melting temperature (°C)
**NKp30**	66/30	N.D.	-3,387	1,000	10^6	87
**NKp44**	72/30	N.D.	-3,501	0,998	10^6	90

NKp30: 1:10 serial dilution of PCR product

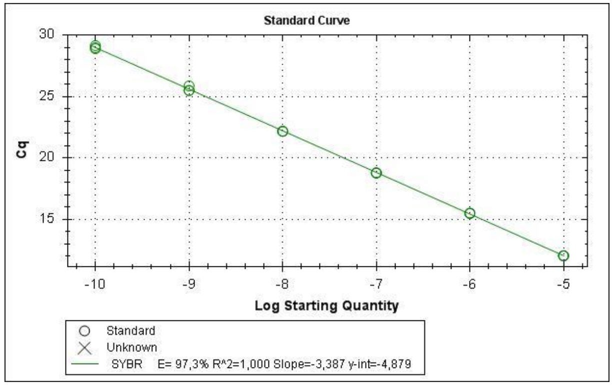

calibration curve

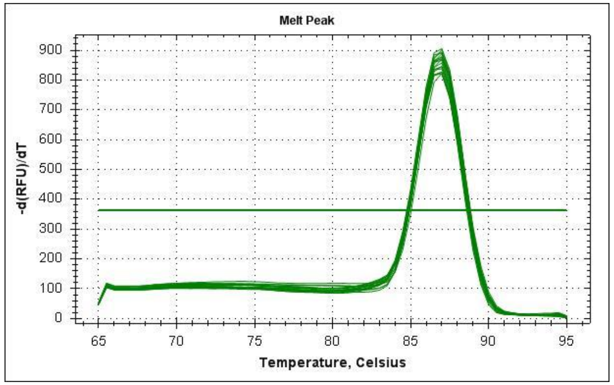

melt curve

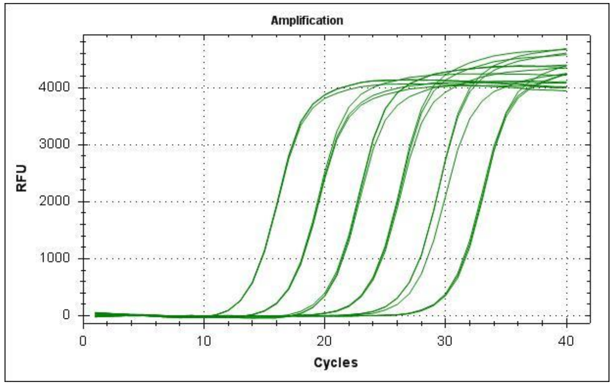

amplification plot

NKp44: 1:10 serial dilution of PCR product

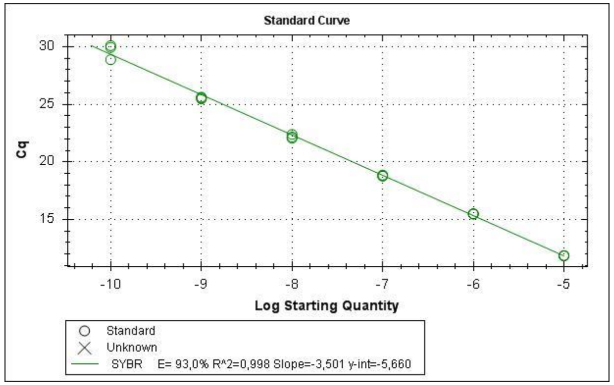

calibration curve

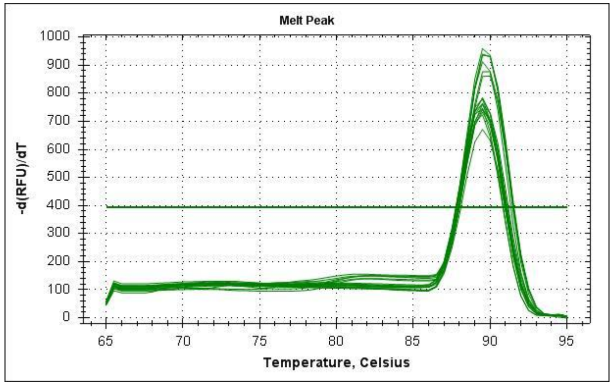

melt curve

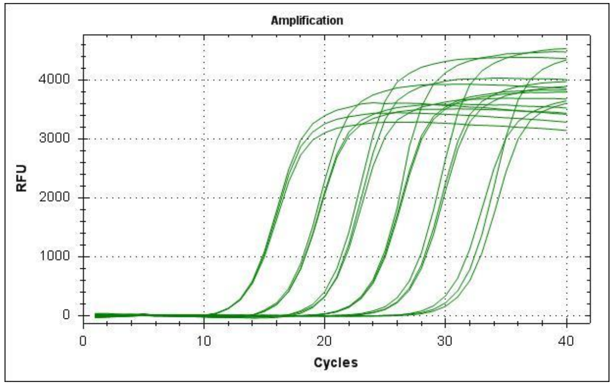

amplification plot
